# Interprofessional education in graduate medical education: survey study of residency program directors

**DOI:** 10.1186/s12909-017-1104-z

**Published:** 2018-01-10

**Authors:** Morhaf Al Achkar, Mathew Hanauer, Chantel Colavecchia, Dean A. Seehusen

**Affiliations:** 1 University of Washington Family Medicine Residency, 331 NE Thornton Place, Seattle, WA 98125 USA; 2 Department of Family Medicine-Indiana University, Indianapolis, Indiana USA; 3 Dwight D. Eisenhower Army Medical Center, Fort Gordon, Georgia USA

**Keywords:** Interprofessional education, Residency, Graduate medical education, Program directors, Survey

## Abstract

**Background:**

The overarching purpose of this study is to examine the current trends in interprofessional education (IPE) within graduate medical education in the Unites States.

**Methods:**

A survey was sent to program directors across with different specialties between March and April 2016. The survey was completed by 233 out of 1757 program directors, which represents a response rate of 13.3%.

**Results:**

IPE is currently being used by over 60% of the GME program directors that completed the survey. The median number of IPE hours is 60. Classroom learning (70.8%) and team-based approaches (70.1%) to patient care are the two most common forms of IPE. The two most prevalent reasons for implementing IPE are improving collaboration (92.2%) and communication (87%). More than half of the program directors agreed or strongly agreed that lack of time both for teachers (54.4) and for residents (51.5%) are barriers to IPE. About one third of the respondents whose programs do not include IPE are interested in implementing some IPE in the future.

**Conclusion:**

IPE in its varying formats has been implemented as a training model by many residency programs. Further studies are needed to explore the comparative effectiveness of the different modalities of IPE.

**Electronic supplementary material:**

The online version of this article (doi:10.1186/s12909-017-1104-z) contains supplementary material, which is available to authorized users.

## Background

Interprofessional education (IPE) in health care is defined as the placement of learners from different health disciplines into an environment where they pursue shared educational goals, learning with one another, from one another, and about each other [1]. IPE aims to foster collaboration among health care professionals from different disciplines so that together they can provide safer, more effective, and more efficient patient care [2]. As such, the Institute of Medicine (IOM) has called for the incorporation of IPE into the training curricula for health care providers as a way to facilitate collaboration between disciplines [3].

Medical errors are the third-leading cause of death in the United States [4], which makes decreasing medical errors one of the field’s top priorities. IPE has been proposed to improve patient safety by simulating emergency situations and providing opportunities for practice. IPE training participants believe their training has enhanced patient outcomes [5]. A 2013 Cochrane review identified 15 studies that measured the effectiveness of IPE interventions. Seven of the identified studies indicated that IPE has positive effects in the following areas: emergency department culture, patient satisfaction, diabetes care, collaborative team behavior in general and in surgeries, error reduction in emergency departments, management of care in domestic violence, and competencies of mental health practitioners in patient care delivery [6].

Since graduate medical education (GME) represents the pipeline for training future physicians, it is not surprising that incorporating IPE into GME has gained increasing interest. IPE in the GME setting often combines residents with members of other health care disciplines, including, but not limited to, the following: residents and faculty members who have other specialties, nurses and nursing students, psychologists and psychology interns, pharmacists, social workers, homecare providers, hospice care providers, clinical lab workers, and medical administrators [7–10]. IPE has been shown to help residents develop the confidence they need to challenge superiors when necessary, which has the potential to reduce medical errors and improve health outcomes [7, 11].

While the literature supports the increased interest in IPE as a model of training for healthcare providers in general, little is known about the trend within GME specifically. Describing the current prevalence and modes of IPE will help direct and prioritize the limited resources and provide groundwork for further research on IPE in the GME setting to understand links between such training, on the one hand, and both learning and healthcare outcomes, on the other. Furthermore, understanding the common barriers to implementing IPE may inform efforts to mitigate these challenges. Finally, exploring the current beliefs among the directors of programs that do not have IPE will help identify ways of encouraging further implementation of IPE in the GME setting.

This study was conducted with the overarching aim of examining the current trends of IPE within GME in the Unites States across several specialties. The study’s specific objectives are threefold: 1) To identify the prevalence and format of, the participants in, and the barriers to IPE; 2) to examine the goals and assessments of IPE experiences; and 3) to explore potential IPE models for programs that do not currently use IPE.

## Methods

### Design

This survey study was part of an omnibus survey sent to all the program directors in the following specialties: family medicine, internal medicine, pediatrics, psychiatry, obstetrics and gynecology, emergency medicine, and surgery. After reviewing the literature, we developed a questionnaire, and then we sought feedback from IPE experts at Indiana University. The Indiana University Institutional Review Board reviewed and approved the questionnaire. The survey questions are provided in Additional file 1. We used RedCap to send the survey to all the program directors listed in the Accreditation Council of Graduate Medical Education (ACGME) residency directories; they received an initial email invitation to participate in the study, followed by three email reminders sent 5 days apart.

### Participants

We used the American Medical Association (AMA) database to identify program directors and obtain their email addresses. We identified 1757 directors and checked 1479 (84.2%) of their email addresses, using program websites to link directly to the program directors’ email addresses. The rest of the email addresses (278, 15.8%) were either generic program or coordinator email addresses. The IPE component of the survey was completed by 233 program directors, which represents a response rate of 13.3%.

### Analysis

To analyze the results, we calculated descriptive statistics of frequencies and percentages. In addition, we used tables and graphs to present the results.

## Results

### Current IPE experiences

**Fig. 1:**
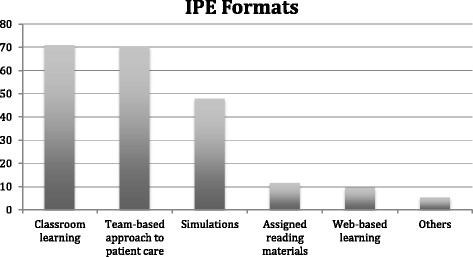
Barriers to IPE

The characteristics of the program directors and their residencies are included in Table 1. Among the respondents, 144 (61.8%) reported having experience with IPE. The total number of hours of IPE varied widely between programs; the median was 60 h. More than half of the program directors agreed or strongly agreed that lack of time both for residents (54.4%) and for teachers (51.5%) were barriers to IPE. Figure 1 shows barriers to IPE as indicated by program directors. The top five barriers were: 1) time for teachers (54.4%), 2) time for residents (51.5%), 3) financial support (33.6%), 4) space to host activities (30.7%), and 5) faculty buy-in (25.2%).

**Table 1:** Residencies’ and program directors’ characteristics and prevalence of IPE

Characteristic	Frequency	Percentage	Has IPE	Percentage
Type of Program				
University-Based Community	102	43.78	67	65.69
Community-Based, University-Affiliated	98	42.06	58	59.18
Community-Based, Non-Affiliated	27	11.59	15	55.56
Military	5	2.15	3	60
Other	1	0.43	1	100
Specialty				
Emergency Medicine	27	11.59	20	74.07
Family Medicine	61	26.18	45	73.77
Internal Medicine	46	19.74	25	54.35
Obstetrics/Gynecology	25	10.73	14	56
Psychiatry	17	7.3	9	52.94
Surgery	22	9.44	12	54.55
Pediatrics	35	15.02	19	54.29
Community Size				
Less than 30,000	3	1.29	2	66.67
30,000 to 74,999	17	7.3	10	58.82
75,000 to 149,999	32	13.73	16	50
150,000 to 499,999	54	23.18	36	66.67
500,000 to 1 million	42	18.03	28	66.67
More than 1 million	85	36.48	52	61.18
Proportion of Non-US Graduates				
0 to 24%	135	57.94	91	67.41
25 to 49%	23	9.87	12	52.17
50 to 74%	23	9.87	15	65.22
75 to 100%	50	21.46	24	48
Don’t Know	1	0.43	1	100
No Answer	1	0.43	1	100
Gender				
Male	141	60.52	85	60.28
Female	90	38.63	58	64.44
No Answer	2	0.86	1	50
Region				
Midwest	64	27.47	41	64.06
Northeast	61	26.18	38	62.3
South	62	26.61	39	62.9
West	35	15.02	20	57.14
No Answer	11	4.72	6	54.55
	Mean	Std. Dev.		
Years Since Program Started	43.43	21.59		
Years as a Program director	6.82	5.87

**Fig. 2:**
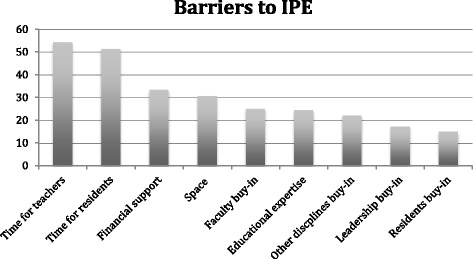
IPE Formats

Classroom learning and team-based approaches to patient care came first and second, respectively, among the forms of IPE and were mentioned in association with 102 (70.8%) and 101 (70.1%) programs. Simulations were used by 69 (47.9%) programs. Web-based learning was used by only 14 (9.7%) programs. Figure 2 represents the IPE formats used by different programs. The duration of the specific IPE experiences mentioned varied widely, with a median of 20 h. IPE was conducted as a single session in 22.0% of the cases and longitudinally in 78.0%. Nursing learners (nurses and nursing students) were the most common participants (59.0%) with whom residents participated in IPE, followed by pharmacy learners (54.9%), residents from other disciplines (52.1%), physician assistant learners (27.8%), and medical administrative staff (22.9%).

### IPE goals and assessments

The top five reasons for engaging in IPE were: “to improve collaboration” (92.2%), 2) “to improve communication” (87%), 3) “to improve patient safety” (82.6%), 4) “to improve health care quality” (79.1%), and 5) “to improve attitudes towards teamwork” (71.3%). The most commonly assessed outcomes were: “skills for working on an interdisciplinary team” (53.9%) followed by “satisfaction with the learning experience” (49.6%), “attitude towards interdisciplinary teamwork” (44.4%), “content-specific knowledge” (32.2%), and “attitudes towards specific content” (33.9%). Outcomes were not assessed in 23.5% of the programs that had IPE.

### Contemplated future IPE experiences

Among the programs that did not have IPE, 28 (32.94%) reported interest in implementing IPE. The duration of the experiences mentioned varied widely as well, with a median of 5 h. The contemplated experiences reported were single learning sessions for 38.46% and longitudinal sessions for 61.54% of the respondents. Nursing learners (nurses and nursing students) were again the most common group with whom IPE was contemplated (71.4%), followed by pharmacy learners (50%), residents from other disciplines (35.7%), nutrition and dietetics learners (25%), and physician assistant learners (21.4%).

The top five reasons for wanting to engage in IPE were: “to improve collaboration” (85.7%), 2) “to improve communication” (78.6%), 3) “to improve patient safety” (75.0%), 4) “to improve attitudes towards teamwork” (75.0%), and 5) “to improve patient care efficiency” (46.4%). All the program directors (100%) indicated that some outcomes of IPE learning would be assessed. The outcomes that respondents wanted to assess were: “skills for working in an interdisciplinary team” (71.4%), “attitudes towards interdisciplinary teamwork” (71.4%), “attitudes towards specific content” (53.6%), “satisfaction with the learning experience” (53.6%), and “knowledge about other disciplines” (53.6%).

## Discussion

To our knowledge, this is the first systematic exploration of medical residents’ experiences in IPE. Current IPE is reported by over half of the program directors who responded to our survey across GME specialties. This finding is encouraging, as IPE can have positive impacts on patient outcomes, adherence rates, patients’ satisfaction, clinical process outcomes, and collaborative behavior [12].

IPE was not implemented equally across the specialties of the respondents to this survey. Emergency medicine and family medicine were the two specialties that most clearly incorporated IPE into their curricula. Over 70% of respondents from these two specialties reported ongoing IPE. This may indicate that these two specialties disproportionately value collaboration and good quality communication with other disciplines. This would not be surprising, given the high degree of interaction with other medical professionals that these two specialties experience daily.

Classroom learning was the most commonly reported method of IPE. This points to a potential area for improvement in IPE because classroom learning may not be the ideal means to learn active skills such as collaboration, teamwork, and communication [13]. Didactic learning is considered passive and does not allow learners to practice and demonstrate skill mastery.

Team-based care and simulations do, however, allow for this type of active engagement and practice. Although the literature is rich in examples of IPE simulation, our study showed that simulation is only the third most common form of IPE. This finding may highlight the gap between IPE research findings and current IPE practices and reveals the need for further work in the area of IPE implementation.

There were few differences in characteristics, goals, and measured outcomes between the programs that had implemented IPE and the programs considering it. It appears as though those interested in IPE have practical and feasible ideas for how to incorporate IPE into their programs. These programs may simply need support in overcoming barriers to the implementation of IPE.

The most significant barriers reported by the program directors responding to this survey are: time, resources, and buy-in, which matches previous studies on barriers to IPE [14, 15]. One way to overcome these barriers is to build IPE organically into the curriculum. It is highly likely that even though every residency program is different, there are parts in which residents interact with other types of medical staff. Therefore, when those occasions occur, the GME programs director could take advantage of the naturally occurring IPE opportunities and provide some IPE instruction to complement that experience.

There is a slight discrepancy in measuring IPE outcomes. While 23.5% of the responding GME program directors that currently implement IPE stated that they did not measure IPE outcomes, 100% of the responding program directors interested in implementing IPE stated that they would measure outcomes. This may be because it is more difficult to measure IPE outcomes in practice than directors anticipate. Without outcome data, program directors implementing IPE would have a difficult time assessing their IPE effectiveness. Future research should focus on how to most efficiently measure IPE outcomes.

This study has its limitations. The response rate was less than 14% and therefore, our results should be seen as preliminary and should be generalized only with caution. It is possible that program directors who were more positively inclined towards IPE were more likely to respond to the survey, leading to an overestimate of the amount of ongoing IPE within GME programs. In addition, the quality of the responses depended upon the program directors’ knowledge of what occurs within their own programs. Some program directors likely overestimated how much IPE their residents receive and in what format, whereas others likely underestimated this. Overall, given that this is the first survey of its kind, the authors believe that these results at least begin to shed the light on important issues with IPE within GME.

Future research should examine how programs have addressed the barriers to IPE. A qualitative study interviewing program directors could study programs as they implement IPE to understand how such barriers are overcome. The findings of future research could be shared with GME programs interested in implementing IPE to begin a dialogue and create opportunities for implementing IPE.

## Conclusion

IPE in its varying formats has been implemented as a training model by many residency programs. Further studies are needed to explore the comparative effectiveness of different IPE models and ways of overcoming common barriers to implementing IPE within the GME arena.

## Additional file

Additional file 1: IPE Survey Questions. (DOCX 81 kb)

## Abbreviations

ACGME: Accreditation council of graduate medical Education
AMA: American medical association
GME: Graduate medical education
IOM: Institute of medicine
IPE: Interprofessional education
