# Supplementary material for: Interprofessional education in graduate medical education: survey study of residency program directors
Source: BMC Med Educ. 2018 Jan 10;18:11. doi: 10.1186/s12909-017-1104-z (PMC5763580; doi:10.1186/s12909-017-1104-z)
Supplement: Additional file 1: — IPE Survey Questions. (DOCX 81 kb) [file 12909_2017_1104_MOESM1_ESM.docx]

# Appendix 1. IPE Survey Questions

In the following section we would like to ask you about interprofessional education (IPE) in your residency. For the purpose of this survey, IPE is defined as placing health care learners from different professional disciplines into an environment where they learn together, from each other, and about one another while pursuing shared education goals.

1. Does your program routinely conduct any form of IPE?

- Yes
- No

Branching here:

If “Yes”, go to 2

If “No”, go to 13

1. Which format(s) of IPE, does your program use? – Select all that apply.

- Interdisciplinary classroom learning (grand round, didactic lectures and seminars, workshops, morbidity, and mortality conference)
- Interdisciplinary simulations (OSCEs, simulation labs, etc.)
- Interdisciplinary team approach to patient care
- Web-based learning
- Assigned reading materials
- Others (Please specify ________________________________)

1. During which year(s) of residency training do your residents participate in IPE? (Please check any responses that apply and indicate the number of hours.)

- First year: yes, no About how many total hours? ______
- Second year: yes, no About how many total hours? ______
- Third year: yes, no About how many total hours? ______
- Fourth year: yes, no, n/a About how many total hours? ______
- Fifth year: yes, no, n/a About how many total hours? ______

1. Rate on a scale 1-5 (1=not important at all, 3=somewhat important, and 5=extremely important) the barriers for implementing IPE at your program

- Lack of financial support
- Lack of educational expertise
- Lack of space to include all the learners
- Lack of buy-in from faculty members
- Lack of buy-in from residents
- Lack of buy-in from other disciplines
- Lack of buy-in from leadership
- Lack of time for teachers
- Lack of time for residents
- Others (Please specify ______)

1. Think about the main IPE activity your residents are currently involved in.  Please describe the IPE activity in 2-3 sentences.
2. With whom do your residents participate in this activity? – Select all that apply.

- Residents from other medical specialties (Please specify ______)
- Nursing learners (nurses or nursing students)
- Medical administrative staff
- Pharmacy learners (pharmacy students or pharmacists)
- Physical therapy learners (physical therapists or physical therapy students)
- Physician assistant (PA) learners (PAs and PA students)
- Public health learners (students and practitioners)
- Nutrition and dietetics learners (students and practitioners)
- Optometry learners (students and practitioners)
- Mental health learners (students and practitioners)
- Others (Please specify ______)

1. Is the activity offered as a single intervention or as a longitudinal training (single answer)?

- Single interventions (such as one-time workshop, single noon conference, etc)
- Longitudinal training

1. About how many total hours? ______(open ended)
2. Which PGY residents participate in this activity (Select all that apply)?

- First year
- Second year
- Third year
- Fourth year
- Fifth year

1. What are the reasons for engaging your residents in this IPE activity? (Select all that apply)

- To improve collaboration
- To improve communication
- To improve attitudes towards teamwork
- To improve patient safety
- To expand residents’ medical knowledge about specific content
- To improve residents skills in infrequently performed tasks and procedures
- To improve residents’ leadership skills
- To improve health care quality
- To improve patient care efficiency
- To improve residents problem solving and decision making
- Others (Please specify _____________________)

1. The outcomes assessed after this IPE activity are (include all that applies)

- Content specific knowledge
- Content specific skills
- Attitudes towards the specific content
- Satisfaction with the learning experience
- Knowledge about other disciplines
- Skills in working within an interdisciplinary teams
- Attitude towards interdisciplinary teamwork
- Attitudes towards other disciplines
- Others (Please specify____________________________________)
- Outcomes were not assessed

1. (if the progoram answers “no” to having IPE) Are you interested in implementing IPE in the future?

- Yes
- No

1. Think about an IPE activity you would like to implement. Please describe this activity in 2-3 sentences.

- IPE activity:______

1. With whom would your residents participate in this proposed activity? – Select all that apply.

- Residents from other medical specialties (Please specify ___________)
- Nursing learners (nurses or nursing students)
- Medical administrative staff
- Pharmacy learners (pharmacy students or pharmacists)
- Physical therapy learners (physical therapists or physical therapy students)
- Physician assistant (PA) learners (PAs and PA students)
- Public health learners (students and practitioners)
- Nutrition and dietetics learners (students and practitioners)
- Optometry learners (students and practitioners)
- Mental health learners (students and practitioners)
- Others (Please specify ___________________________________)

1. Would the activity be offered as a single intervention or as a longitudinal training (single answer)?

- Single interventions
- Longitudinal training

1. About how many total hours? ______
2. Which PGY residents would participate in this activity (Select all that apply)?

- First year
- Second year
- Third year
- Fourth year
- Fifth year

1. What are the reasons for engaging your residents in this proposed IPE activity? (Select all that apply)

- To improve collaboration
- To improve communication
- To improve attitudes towards teamwork
- To improve patient safety
- To expand residents’ medical knowledge about specific content
- To improve residents skills in infrequently performed tasks and procedures
- To improve residents’ leadership skills
- To improve health care quality
- To improve patient care efficiency
- To improve residents problem solving and decision making
- Others (Please specify __________________________________________)

1. The outcomes will be assessed after this IPE activity would be (include all that applies)

- Content specific knowledge
- Content specific skills
- Attitudes towards the specific content
- Satisfaction with the learning experience
- Knowledge about other disciplines
- Skills in working within an interdisciplinary teams
- Attitude towards interdisciplinary teamwork
- Attitudes towards other disciplines
- Others (Please specify______________________________________________)
- Outcomes will not be assessed
